# Absenteeism among medical and health science undergraduate students at Hawassa University, Ethiopia

**DOI:** 10.1186/1472-6920-14-81

**Published:** 2014-04-14

**Authors:** Anteneh Assefa Desalegn, Asres Berhan, Yifru Berhan

**Affiliations:** 1 Pharmacology Unit, School of Medicine, Hawassa University, P.O. Box-1560, Hawassa, Ethiopia; 2 Department of gynecology and obstetrics, School of Medicine, Hawassa University, Hawassa, Ethiopia

**Keywords:** Non-attendance, Absenteeism, Lecture, Tutorial, University students

## Abstract

**Background:**

Student absenteeism is a major concern for university education worldwide. This study was conducted to determine the prevalence and causes of absenteeism among undergraduate medical and health sciences students at Hawassa University.

**Methods:**

We conducted a cross-sectional study using a pretested self-administered structured questionnaire from May-June 2013. The primary outcome indicator was self-reported absenteeism from lectures in the semester preceding the study period. The study included all regular undergraduate students who were enrolled in the University for at least one semester. The data was entered and analyzed using SPSS version 20. The association between class absenteeism and socio-demographic and behavioral correlates of absenteeism was determined by bivariate and multivariate analyses. Results were reported as crude odds ratios (COR), adjusted odds ratios (AOR) and 95% confidence intervals (CI).

**Results:**

1200 students consented and filled the questionnaire. Of these students, 43.7% had missed three or more lectures and 14.1% (95% CI = 12.2-16.2) missed more than 8 lectures in the preceding semester. There was a significant association between missing more than 8 lectures and age of students, chosen discipline (medicine), and social drug use. The main reasons reported for missing lectures were preparing for another examination, lack of interest, lecturer’s teaching style, and availability of lecture material.

**Conclusion:**

At Hawassa University College of Medicine and Health Science student habits and teacher performance play a role in absenteeism from lectures. A university culture that promotes discipline and integrity especially among medical and older students discourages social drug use will likely improve motivation and attendance. Training in teaching methodologies to improve the quality and delivery of lectures should also help increase attendance.

## Background

Student absenteeism at lectures is a major concern in Hawassa University. Many higher institutions in Ethiopia, including Hawassa University, have explicit policies regarding mandatory attendance during lecture, laboratory and practical sessions. Despite the strict rules, absenteeism is an on-going problem in Ethiopian universities; a phenomenon that is also on the rise in universities worldwide [1-3].

Absenteeism has been shown to be an indicator of low level of motivation for learning [1]. There is extensive literature on the link between absenteeism and lack of subject matter interest, poor teaching strategies, unfavorable learning environment, excessive socialization among students, part-time jobs, ill health, sleeplessness, and poor relations with lecturers [1,2,4-6]. In addition, accessibility of lecture content in the form of online slides, videos, audios have their own contribution to absenteeism [7-11].

A meta-analysis consisting 52 published articles and 16 unpublished papers on the impact of absenteeism on students’ academic performance found a negative association [12]. Other studies have also re-affirmed the indispensable contribution of attending class to better knowledge and cumulative grade point average (CGPA) [13-18]. However, studies have also disputed the role of regular class attendance for better academic performance by emphasizing the importance of prior CGPA, motivation and a score in scholastic aptitude test (SAT) for better academic performance [2,19]. Furthermore, some argue that not all students learn best from a “didactic lecture” scenario, especially if someone isn’t an auditory learner [20].

The reasons for student absenteeism are similar, though the level and magnitude of each reason may differ from country to country. Despite the observed nature of the problem (absenteeism from class in higher education), there is no published study that assessed the magnitude and predictors of absenteeism in any Ethiopian University. Thus, this study was conducted to determine the magnitude and predictors of absenteeism among undergraduate students of Hawassa University College of Medicine and Health Sciences.

## Methods

### Study design and setting

We conducted a cross-sectional study on class attendance of undergraduate students at Hawassa University College of Medicine and Health Sciences using a self-administered questionnaire. The College of medicine and health sciences was established in 2003 and consists of three schools (Medicine; Public health and Environmental sciences; Nursing and Midwifery) and the department of Medical Laboratory Sciences with 7 undergraduate programs.

### Eligibility criteria and data collection

The study included all full-time undergraduate students who were enrolled in the college for at least one semester and gave consent to participate in the study. The questionnaire was pre-tested on part-time undergraduate health science students. In parts of the questionnaire that were found to be misleading or confusing, a slight modification was made on the wording before data collection. Data collection was done between May and June, 2013. Sealed envelopes containing the pre-tested questionnaires were distributed to study participants by trained data collection facilitators. To ensure anonymity, no identifying information was included in the questionnaire and the participants were asked to deposit the sealed envelopes in a data collection box.

### Measurement of class absenteeism

The primary outcome indicator (dependent variable) was self-reported class attendance during the preceding semester. The independent variables included student sex, age, religion, place of accommodation while at the university, type of high school completed (public or private), monthly income, parents’ educational level, field of study, year of study, and reasons for missing classes.

### Statistical analysis

We conducted analysis after dichotomizing class absenteeism in three ways: never missed lectures vs. missed at least one lecture during the preceding semester; missed less than 3 lectures vs. missed 3 or more lectures in the preceding semester; and missed at least 8 lectures vs. missed more than 8 lectures in the preceding semester. The dichotomization was done to simplify the analysis and interpretation of results. The association between class absenteeism and socio-demographic and other variables was determined using bivariate and multivariate analysis model and reported as unadjusted odds ratios (OR) and adjusted odds ratios (AOR) with 95% confidence intervals (CI), respectively. After testing for colinearity and interaction, we calculated the adjusted OR (AOR) by including covariates with a p-value ≤ 0.1 in the multivariate analysis. All the statistical tests were two tailed and considered statistically significant for p-value < 0.05 and the 95% CI did not contain the number 1. The data were entered, cleaned, coded, and analyzed using SPSS version 20 software.

### Ethical clearance

Ethical clearance was obtained from Institutional review board (IRB) of Hawassa University College of Medicine and Health Sciences. We obtained written consent from each study participant, and maintained confidentiality by analyzing the data in aggregate.

## Results

Of the 1,366 eligible full-time undergraduate students, 1,220 (89%) consented and completed the questionnaire. The main reason for non-participation was unavailability of students in lecture halls, classrooms or dormitories during the survey due to clinical or community based attachments outside the University. The mean age of the students surveyed was 21.5 years (SD = 1.902 years) and the average number of students per class is 83.4 (SD = 46.8), with a minimum of 14 students per class and a maximum of 255 students per class. The demographic characteristic of the study participants is presented in Table 1.

**Table 1:** Baseline characteristics of students

**Socio-demographic variables**		**Number of students who never missed class per semester (%)**	**Number of students who had missed class 1 to 3 times per semester (%)**	**Number of students who had missed class 4 times and above per semester (%)**	**Total**
Sex	Male	248 (25.8)	376 (39.2)	336 (35.0)	960 (78.8%)
	Female	51 (19.7)	107 (41.3)	101 (39.0)	259 (21.2%)
Where they grew up	Urban area	137 (20.1)	247 (36.2)	299 (43.8)	683 (57.7%)
	Rural area	150 (29.9)	220 (43.9)	131 (26.1)	501 (42.3%)
Religion	Orthodox	174 (24.6)	280 (39.5)	254 (35.9)	708 (58.4%)
	Protestant	88 (27.4)	127 (39.6)	106 (33.0)	321 (26.4%)
	Muslim	23 (16.9)	60 (44.1)	53 (39.0)	136 (11.2%)
	Other	9 (18.8)	16 (33.3)	23 (47.9)	48 (4%)
High school	Public school	263 (27.6)	389 (40.8)	301 (31.6)	953 (79.1%)
	Private school	24 (10.6)	87 (38.3)	116 (51.1)	227 (18.8%)
	Missionary school	6 (23.1)	3 (11.5)	17 (65.4)	26 (2.2%)
University entrance examination score range	Excellent	42 (17.4)	89 (36.8)	111 (45.9)	242 (21.5%)
	Very good	140 (26.6)	215 (40.8)	172 (32.6)	527 (47%)
	Good	83 (25.9)	141 (43.9)	97 (30.2)	321 (28.5%)
	Fair	6 (27.3)	8 (36.4)	8 (36.4)	22 (2%)
	Poor	3 (25.0)	2 (16.7)	7 (58.3)	12 (1%)
Students mother education level	No education	112 (27.3)	181 (44.0)	118 (28.7)	411 (34.4%)
	Primary	90 (27.2)	130 (39.3)	111 (33.5)	331 (27.7%)
	Secondary	44 (22.0)	75 (37.5)	81 (40.5)	200 (16.7%)
	Diploma	24 (15.8)	50 (32.9)	78 (51.3)	152 (12.6%)
	Degree	21 (20.6)	40 (39.2)	41 (40.2)	102 (8.5%)
Student’s father education level	No education	82 (27.2)	132 (43.9)	87 (28.9)	301 (25.2%)
	Primary	84 (28.7)	121 (41.3)	88 (30.0)	293 (24.5%)
	Secondary	44 (23.0)	83 (43.5)	64 (33.5)	191 (16%)
	Diploma	35 (20.3)	59 (34.3)	78 (45.3)	172 (14.3%)
	Degree	45 (18.8)	86 (36.0)	108 (45.2)	239 (20%)
Current residence	In the university’s dormitory	271 (24.2)	452 (40.3)	398 (35.5)	1121 (92.6%)
	Outside the campus at rental house	19 (33.9)	21 (37.5)	16 (28.6)	56 (4.6%)
	Outside the campus with family	7 (21.2)	9 (27.3)	17 (51.5)	33 (2.7%)
Money they get monthly from family or relatives	Less than 100 birr	36 (31.6)	49 (43.0)	29 (25.4)	114 (9.6%)
	100-200 birr	56 (25.9)	104 (48.1)	56 (25.9)	216 (18.1%)
	200-300 birr	80 (29.6)	105 (38.9)	85 (31.5)	270 (22.6%)
	300-500 birr	96 (23.2)	164 (39.7)	153 (37.1)	413 (34.6%)
	More than 500 birr	26 (14.4)	56 (31.1)	98 (54.4)	180 (15.1%)
Place to have meal	At the university’s cafeteria (cafe)	258 (26.6)	391 (40.3)	321 (33.1)	970 (80.4%)
	Non-café	38 (16.1)	89 (37.7)	109 (46.2)	236 (19.6%)
Field of study	Medicine	41 (9.6)	131 (30.8)	253 (59.5)	425 (35.1%)
	Nursing	58 (30.7)	90 (47.6)	41 (21.7)	189 (15.7%)
	Midwifery	38 (31.4)	55 (45.5)	28 (23.1)	121 (10%)
	Medical laboratory technology	36 (37.5)	39 (40.6)	21 (21.9)	96 (8%)
	Health officer	97 (34.6)	117 (41.8)	66 (23.6)	280 (23.2%)
	Optometry	15 (24.2)	30 (48.4)	17 (27.4)	62 (5.1%)
	Environmental	11 (31.4)	20 (57.1)	4 (11.4)	35 (2.9%)
Joined the field of study as per choice	Yes	202 (23.5)	334 (38.8)	325 (37.7)	861 (70.5%)
	No	96 (27.4)	149 (42.5)	106 (30.2)	351 (29.5%)
Like their field of study	Yes	229 (25.3)	363 (40.1)	313 (34.6)	905 (75%)
	No	67 (22.1)	118 (38.9)	118 (38.9)	303 (25%)
Year of study	1st year	51 (37.2)	70 (51.1)	16 (11.7)	137 (11.3%)
	2nd year	86 (24.1)	167 (46.8)	104 (29.1)	357 (29.4%)
	3rd year	66 (23.2)	111 (38.9)	108 (37.9)	285 (23.4%)
	4th year	83 (23.6)	109 (31.1)	159 (45.3)	351 (28.9%)
	5th year	13 (14.9)	26 (29.9)	48 (55.2)	87 (7.2%)
Social drug use	Khat	13 (19.4)	25 (37.3)	29 (43.3)	67 (5.7%)
	Cigarette	5 (26.3)	3 (15.8)	11 (57.9)	19 (1.6%)
	Shisha	2 (15.4)	0 (0.0)	11 (84.6)	13 (1.1%)
	Alcohol	11 (23.9)	19 (41.3)	16 (34.8)	46 (3.9%)
	Marijuana/hashish	1 (8.3)	2 (16.7)	9 (75.0)	12 (1%)
	Never used	256 (25.4)	420 (41.6)	333 (33.0)	1009 (86.5%)

Of the students who participated in the study, 75% had missed one or more classes and 45% had missed three or more classes in the preceding semester. The odds of students who missed at least one class in the preceding semester and who had grown up in rural area was 40% less than students who had grown up in urban areas (OR = 0.6; 95% CI, 0.45 to 0.77). In the multivariate analysis, there was no association between student’s age, sex, religion, and father’s education level and missing at least one class in the preceding semester (Table 2). On the other hand, we noted an association between mother’s education level and absenteeism with the odds of missing one or more classes per semester being 76% less in students whose mother had a university degree (adjusted OR = 0.26, 95% CI = 0.09-0.75) as compared to those whose mother was illiterate (Table 2).

**Table 2:** Odds ratio of variables (never missed class vs. missed at least once per semester)

**Variables**		**P-value**	**Unadjusted ORs (95% CI)**	**P-value**	**AOR (95% CI)**
Sex	Male (ref)				
	Female	0.042	1.42 (1.013, 1.993)	0.846	1.06 (0.594, 1.889)
Grown up	Urban (ref)				
	Rural	<0.0001	0.59 (.449, 0.767)	0.20	0.750 (0.483, 1.164)
Religion	Orthodox (ref)				
	Protestant	0.333	0.86 (0.640, 1.163)	0.439	0.84 (0.544, 1.303)
	Muslim	0.055	1.60 (0.991, 2.587)	0.225	1.51 (0.775, 2.951)
	Other religion	0.364	1.41 (0.671, 2.973)	0.834	0.89 (0.304, 2.612)
High school	Governmental or public (ref)				
	Private	<0.0001	3.22 (2.063, 5.037)	**0.010**	2.49 (1.246, 4.959)
	Missionary	0.611	1.27 (0.505, 3.199)	0.995	1.00 (0.219, 4.517)
University entrance examination score range	Excellent (ref)				
	Very good	0.006	0.58 (0.395, 0.853)	0.872	0.96 (0.562, 1.629)
	Good	0.017	0.60 (0.397, 0.913)	0.491	1.23 (0.687, 2.189)
	Fair	0.254	0.56 (0.207, 1.515)	0.522	1.71 (0.331, 8.848)
	Poor	0.502	0.630 (0.164, 2.426)	0.781	1.29 (0.211, 7.900)
Students mother or next of kin education level	No education (ref)				
	Primary	0.985	1.00 (0.725, 1.389)	0.297	0.75 (0.433, 1.292)
	Secondary	0.163	1.33 (0.891, 1.979)	0.051	0.46 (0.210, 1.004)
	Diploma	0.005	2.00 (1.228, 3.251)	0.063	0.42 (0.171, 1.049)
	Degree	0.171	1.45 (0.853, 2.447)	**0.013**	0.26 (0.089, 0.752)
Students father or next of kin education level	No education (ref)				
	Primary	0.699	0.93 (0.651, 1.333)	0.564	1.18 (0.671, 2.077)
	Secondary	0.298	1.25 (0.821, 1.907)	0.158	1.71 (0.812, 3.603)
	Diploma	0.096	1.47 (0.935, 2.298)	0.266	1.57 (0.709, 3.479)
	Degree	0.023	1.614 (1.069, 2.437)	0.103	2.06 (0.864, 4.925)
Place to have meal	In the university cafeteria (ref)				
	Non-café	0.001	1.89 (1.297, 2.748)	0.180	1.56 (0.814, 3.002)
Money they get monthly from family or relatives	Less than 100 birr (ref)				
	100-200 birr	0.277	1.32 (0.801, 2.171)	0.958	1.02 (0.502, 2.070)
	200-300 birr	0.704	1.10 (0.683, 1.760)	0.600	0.83 (0.419, 1.653)
	300-500 birr	0.070	1.52 (0.966, 2.405)	0.819	0.92 (0.459, 1.852)
	More than 500 birr	0.001	2.73 (1.541, 4.850)	0.399	1.48 (0.596, 3.669)
Field of study	Medicine (ref)				
	Nursing	<0.0001	0.24 (0.154, 0.377)	**<0.0001**	0.20 (0.088, 0.428)
	Midwifery	<0.0001	0.23 (0.141, 0.385)	**0.001**	0.24 (0.098, 0.567)
	Medical laboratory technology	<0.0001	0.18 (0.105, 0.300)	**<0.0001**	0.14 (0.051, 0.388)
	Health officer	<0.0001	0.20 (0.134, 0.302)	**<0.0001**	0.19 (0.099, 0.369)
	Optometry	0.001	0.34 (0.172, 0.650)	**0.003**	0.16 (0.047, 0.536)
	Environmental	<0.0001	0.23 (0.106, 0.510)	**0.008**	0.17 (0.046, 0.626)
Year of study	1st year (ref)				
	2nd year	0.004	1.87 (1.224, 2.852)	0.398	1.32 (0.690, 2.541)
	3rd year	0.003	1.97 (1.264, 3.063)	0.583	1.20 (0.627, 2.297)
	4th year	0.003	1.92 (1.252, 2.929)	0.080	1.84 (0.929, 3.657)
	5th year	<0.0001	3.38 (1.704, 6.688)	0.549	0.71 (0.233, 2.171)
Total number of students in a class (per one additional student)		<0.0001	1.01 (1.005, 1.011)	0.257	1.00 (0.989, 1.003)
Reason for missing classes	Class schedule inconvenience	<0.0001	7.78 (5.320, 11.369)	**<0.0001**	13.72 (8.456, 22.244)
	The subject does not need teachers guidance (simplicity)	<0.0001	3.33 (1.849, 5.989)	**<0.0001**	8.10 (3.942, 16.640)
	Lack of interest to the subject	<0.0001	5.14 (2.057, 12.830)	**<0.0001**	9.15 (3.359, 24.926)
	Do not like teachers teaching style	<0.0001	5.24 (3.285, 8.344)	**<0.0001**	3.37 (1.877, 6.032)

Note: P-values in bold are statistically significant (p < 0.05).

The odds of missing one or more classes per semester was 3 times higher among students who completed their high school study in private schools as compared to those who completed their high school in public school (OR = 3.2; 95% CI, 2.06 to 5.04). Relative to students who got “excellent grade” in the university entrance examination, students who scored in the range of “very good grade” and “good grade” were more likely to miss one or more classes. In the multivariate analysis model, the odds of missing at least one or more class in a semester was about 2.5 times higher among students who completed their high school study in private schools (AOR = 2.5; 95% CI, 1.25 to 4.96) (Table 2).

In the bivariate analysis model, current residence, interest in the field of study, and current social drug use were not associated with class absenteeism. On the other hand, students in a large class size (OR = 1.0; 95% CI, 1.005 to 1.011), students whose income was more than 25 USD per month (OR = 2.7, 95% CI, 1.54 to 4.85), and did not have meal in the university cafeteria (OR = 1.9; 95% CI, 1.30 to 2.75) were significantly associated with missing at least one or more classes per semester. Relative to first year students, 2nd, 3rd, 4th and 5th year students were more likely to miss one or more classes in a semester. Surprisingly, the odds of missing one or more classes per semester among medical students was 75% more than among health science students (Table 2).

Missing 3 or more classes was higher in older students (OR = 1.2; 95% CI; 1.10 to 1.26), and students with a better educated mothers and fathers. Among students who grew up in rural areas, the odds of missing 3 or more classes per semester was 54% less than students who grew up in urban areas (OR = 0.5; 95% CI, 0.36 to 0.58). Missing three or more classes per semester was not associated with sex of the student or their religion. In the multivariate analysis model, the odds of not missing 3 or more classes per semester remained strongly associated with students who grew up in rural areas (AOR = 0.4; 95% CI, 0.25 to 0.66) (Table 3).

**Table 3:** Odds ratio of variables (missed class <3 times vs missed class ≥3 per semester)

		**P-value**	**Unadjusted ORs (95% CI)**	**P-value**	**AOR (95% CI)**
Age		<0.0001	1.18 (1.102, 1.257)	**0.020**	1.19 (1.028, 1.374)
Grown up	Urban (ref)				
	Rural	<0.0001	0.46 (0.360, 0.579)	**<0.0001**	0.41 (0.251, 0.663)
High school	Governmental or public (ref)				
	Private	<0.0001	2.56 (1.897, 3.453)	**0.030**	2.10 (1.077, 4.103)
	Missionary	0.005	3.32 (1.429, 7.711)	0.126	3.84 (0.686, 21.496)
University entrance examination score range	Excellent (ref)				
	Very good	<0.0001	0.41 (0.303, 0.565)	0.107	0.645 (0.378, 1.100)
	Good	<0.0001	0.40 (0.283, 0.561)	0.130	0.628 (0.344, 1.147)
	Fair	0.291	0.62 (0.260, 1.497)	0.860	0.843 (0.127, 5.595)
	Poor	0.822	0.87 (0.269, 2.834)	0.613	0.600 (0.083, 4.348)
Students mother or next of kin education level	No education (ref)				
	Primary	0.045	1.35 (1.007, 1.821)	0.337	1.37 (0.723, 2.579)
	Secondary	<0.0001	1.87 (1.326, 2.630)	0.969	0.98 (0.421, 2.297)
	Diploma	<0.0001	2.62 (1.791, 3.842)	0.238	0.530 (0.185, 1.520)
	Degree	0.002	1.98 (1.277, 3.065)	0.419	0.62 (0.197, 1.964)
Students father or next of kin education level	No education (ref)				
	Primary	0.739	0.95 (0.679, 1.316)	0.476	0.78 (0.400, 1.534)
	Secondary	0.313	1.21 (0.836, 1.747)	0.984	0.99 (0.431, 2.283)
	Diploma	0.001	1.90 (1.297, 2.769)	0.553	0.76 (0.313, 1.863)
	Degree	<0.0001	1.97 (1.398, 2.785)	0.587	0.76 (0.287, 2.027)
Place to have meal	In the university cafeteria (ref)				
	Non-café	0.001	1.65 (1.242, 2.202)	0.498	1.27 (0.638, 2.522)
Money they get monthly from family or relatives	Less than 100 birr (ref)				
	100-200 birr	0.326	1.28 (0.783, 2.083	**0.037**	2.50 (1.058, 5.900)
	200-300 birr	0.044	1.62 (1.012, 2.586)	**0.016**	2.92 (1.221, 6.969)
	300-500 birr	<0001	2.28 (1.457, 3.550)	**0.014**	3.00 (1.251, 7.209)
	More than 500 birr	<0001	3.70 (2.241, 6.101)	0.060	2.71 (0.961, 7.618)
Field of study	Medicine (ref)				
	Nursing	<0001	0.17 (0.113, 0.241)	**<0.0001**	0.11 (0.044, 0.253)
	Midwifery	<0001	0.19 (0.122, 0.292)	**0.001**	0.20 (0.075, 0.514)
	Medical laboratory technology	<0001	0.20 (0.122, 0.316)	**0.005**	0.21 (0.073, 0.624)
	Health officer	<0001	0.19 (0.136, 0.262)	**<0.0001**	0.12 (0.061, 0.239)
	Optometry	<0001	0.23 (0.129, 0.397)	**0.003**	0.13 (0.033, 0.487)
	Environmental	<0001	0.17 (0.077, 0.353)	**0.002**	0.11 (0.026, 0.427)
Year of study	1st year (ref)				
	2nd year	<0001	2.66 (1.660, 4.261)	0.335	1.80 (0.545, 5.925)
	3rd year	<0001	3.67 (2.267, 5.931)	0.379	1.73 (0.511, 5.856)
	4th year	<0001	4.65 (2.903, 7.435)	0.352	1.81 (0.518, 6.354)
	5th year	<0001	8.56 (4.636, 15.895)	0.745	1.30 (0.267, 6.326)
Joined field of study as per choice	Yes (ref)				
	No	0.017	0.74 (0.571, 0.946)	0.211	1.40 (0.828, 2.357)
Total number of students in a class (per one additional student)		<0001	1.01 (1.009, 1.014)	0.619	1.00 (0.995, 1.009)
Reason for missing classes	Class schedule inconvenience	<0001	1.93 (1.526, 2.426)	**<0.0001**	3.40 (2.137, 5.405)
	The subject does not need teachers guidance (simplicity)	<0001	3.10 (2.105, 4.561)	**<0.0001**	4.80 (2.403, 9.575)
	Lack of interest to the subject	0.002	2.07 (1.296, 3.312)	0.087	2.19 (0.893, 5.352)
	Do not like teachers teaching style	<0001	2.74 (2.077, 3.611)	**0.014**	1.83 (1.133, 2.954)
First year GPA		0.065	1.33 (0.982, 1.810)	**0.020**	0.52 (0.298, 0.903)
Social drug use	Never used (ref)				
	Khat	0.195	1.39 (0.846, 2.276)	0.847	1.09 (0.465, 2.539)
	Cigarette	0.031	2.92 (1.100, 7.737)	0.392	2.47 (0.311, 19.619)
	Alcohol	0.324	1.35 (0.745, 2.432)	0.159	2.09 (0.750, 5.832)

Note: P-values in bold are statistically significant (p < 0.05).

Students who attended private high schools (OR = 2.6; 95% CI, 1.90-3.45) and missionary high schools (OR = 3.3; 95% CI, 1.49 to 7.71) were significantly associated with missing three or more classes per semester. Students who scored in the university entrance examination in the range of “very good” and “good grade” were also significantly associated with missing 3 or more classes in a semester. In the multivariate analysis model, private high school attendance was an independent predictor for missing 3 or more classes (AOR = 2.1; 95% CI, 1.08 to 4.10) (Table 3).

Missing 3 or more class per semester was also significantly associated with students who had meals outside the university cafeteria; students who got more than 10 USD per month; students who were studying medicine; were in second year and above in seniority; and had a good first year GPA. Large class size and inconvenient class schedule; lack of interest in the subject, teaching style of the teacher and simplicity of the subject matter were also associated with students missing 3 or more classes per semesters. The multivariate analysis also showed a strong association of students who were studying medicine, earning 10 to 25 USD per month, having better first year GPAs, having inconvenient class schedule, disliking the teaching style of the teacher, and lacking of interest in the subject matters with missing 3 or more classes in a semester (Table 3).

The odds of missing more than 8 classes per semester was more than four times higher among students who were 20 years old or older (adjusted OR = 4.20, 95% CI = 1.48-11.92). Consistently, medical students were five times more likely to miss 8 classes per semester as compared with health science students (adjusted OR = 5.02, 95% CI = 1.27-19.73). Social drug use was also very strongly associated with missing more than 8 lectures (adjusted OR = 18.7, 95% CI = 4.59-76.18) (Table 4).

**Table 4:** Socio-demographic and behavioural correlates of absenteeism (missing more than 8 lecture days) among Hawassa University College of medicine and health science students, June 2013

**Factors**	**Missing >8 lecture class days**		**Adjusted OR (95% CI)**	**p-value**
	**No**	**Yes**		
**Age (N = 379)**				
15-19	125 (92.6%)	10 (7.4%)	1.00 (Reference)	**0.007**
20 or more	210 (86.1%)	34 (13.9%)	4.20 (1.48-11.92)	
**Original background (N = 1153)**				
Urban	542 (81.7%)	121 (18.3%)	1.00 (Reference)	0.461
Rural	448 (91.4%)	42 (8.6%)	0.65 (0.21-2.01)	
**Type of high school attended (N = 1174)**				
Public high school	825 (88.9%)	103 (11.1%)	1.00 (Reference)	0.869
Private high school	167 (75.9%)	53 (24.1%)	1.09 (0.36-3.26)	
Missionary high school	16 (61.5%)	10 (38.5%)	0.00	0.999
**Entrance exam score range (N = 1097)**				
Excellent	184 (78%)	52 (22%)	1.00 (Reference)	
Very good	455 (87.8%)	63 (12.2%)	0.54 (0.16-1.75)	0.309
Good	288 (93.2%)	21 (6.8%)	0.68 (0.19-2.47)	0.568
Fair	17 (77.3%)	5 (22.7%)	0.64 (0.05-7.89)	0.729
Poor	7 (58.3%)	5 (41.7%)	0.72 (0.04-11.23)	0.819
**Mother’s education level (N = 1169)**				
Illiterate	363 (91.4%)	34 (8.6%)	1.00 (Reference)	0.115
Literate	639 (82.8%)	133 (17.2%)	4.14 (0.70-24.25)	
**Father’s education level (N = 1171)**				
Illiterate	265 (90.4%)	28 (9.6%)	1.00 (Reference)	0.950
Literate	742 (84.5%)	136 (15.5%)	0.94 (0.16-5.41)	
**Dining place of students (N = 1180)**				
At the university’s café	830 (87.6%)	118 (12.4%)	1.00 (Reference)	0.793
Outside the university café	183 (78.9%)	49 (21.1%)	0.84 (0.24-2.97)	
**Monthly Pocket money (N = 1168)**				
Less than birr 300			1.00 (Reference)	0.085
More than birr 300			2.62 (0.87-7.90)	
**Field of study (N = 1182)**				
Other health sciences	727 (94.3%)	44 (5.7%)	1.00 (Reference)	**0.021**
Medicine	289 (70.3%)	122 (29.7%)	5.02 (1.27-19.73)	
**Year of study (N = 1187)**				0.330
1st year	132 (96.4%)	5 (3.6%)	1.00 (Reference)	
2nd year and above	888 (84.6%)	162 (15.4%)	0.45 (0.09-2.22)	
**Like their field of study (N = 1182)**				
Yes	776 (87.5%)	111 (12.5%)	1.00 (Reference)	0.608
No	239 (81%)	56 (19%)	1.32 (0.45-3.82)	
**Class size (N = 1187)**				
Small	505 (79.9%)	127 (20.1%)	1.00 (Reference)	0.647
Large	515 (92.8%)	40 (7.2%)	0.71 (0.16-3.011)	
**Cumulative GPA (N = 1187)**				
Low	193 (81.8%)	43 (18.2%)	1.00 (Reference)	0.428
High	827 (87%)	124 (13%)	0.55 (0.12-2.38)	
**Use of substance (N = 1144)**				
No	871 (87.6%)	123 (12.4%)	1.00 (Reference)	**<0.0001**
Yes	111 (74%)	39 (26%)	18.70 (4.59-76.18)	

Note: P-values in bold are statistically significant (p < 0.05).

## Discussion

In this study, nearly half of the students, 43.7% (95% CI = 7.8-11.4) reported that they had missed three or more lectures and 14.1% (95% CI = 12.2-16.2) missed more than 8 lectures in the preceding semester. Student absenteeism was significantly associated with age, degree pursued (medical vs. health science) and social drug use. The major reasons reported by the study participants for missing lectures were preparing for another examination, inconvenient class schedule, lack of interest in the subject matter, dislike of teaching style and the ease of understanding the subject matter without guidance. The prevalence of absenteeism and associated risk factors at Hawassa University College of Medicine and Health Sciences were consistent with finding from three South African universities [1]. Studies indicate that absenteeism from lectures and tutorials is a growing trend [7,21-23].

Previous studies have shown that academic and non-academic workload on students could be a factor in affecting attendance [22,1]. University instructors can encourage positive attitudes towards the importance of class attendance [3]. Individual attitude and motivation for learning was a key factor in student absenteeism [22-24,3]. Among our study participants, lack of interest in the subject matter was reported as a reason for non-attendance. This could be attributed to two reasons: 1) the likelihood that the majority of health science students are assigned to the field probably against their choice; 2) even though medical students elect to be on the medical track, they may find the course work more rigorous and demanding.

Quality of lecture as perceived by the student was also found to be a significant factor for very low levels of attendance among students in different studies [22,21,7,3]. More training should be given to instructors in order to improve the teaching methodologies could help improve attendance.

Older students were found more likely to be absent more than 8 lectures in a semester than younger students. This can be attributed to a behavior change due to peer influence and increased level of familiarity with the program and culture on campus. Interventions targeting the younger students might be successful in improving class attendance and behavior during the senior years.

The surprising finding of this study was the strong association of class absenteeism with being a medical student. The medical students were more likely to miss classes than health science students (Nursing, Health Officer, Midwifery, Laboratory Technology, Optometry, and Environmental Health) despite the medical curriculum requirement of 100% class attendance unless the student has legitimate reasons for missing class. This could explain why academic delays and dismissals from the program are commonly observed among medical students, particularly during the final years in this medical school. If this behavior continues after school, it will reflect on their work life and therefore, may affect the quality of health service in the future [12-14,1].

Social drug use was very strongly associated with being absent from lectures. Previous studies have shown that substance abuse [25] and alcohol consumption [26] are risk factors for prevalent and unexcused absence from lectures. Additionally, students with low motivation are more likely to engage in social drug use [27]. Low motivation to learn was significantly associated with being absent in more lectures [2,22,23].

Contrary to other studies, we didn’t find a clear and significant association between absenteeism and academic achievement among the study participants [28,24,22,12-19,2]. Further, our data suggest that class size, background of students, amount of monthly allowance, parental education level, and dining place of students were not significantly associated with missing more lectures.

One of our limitations in assessing the question of student absenteeism is the study design. It is a descriptive cross-sectional study without a qualitative component that does not allow for gathering detailed information to establish causality between absenteeism and its predictors. The data were collected based on self-reporting of non-attendance, which can be subject to recall bias. In addition, under reporting of non-attendance is also probable due to social desirability bias. On the other hand, most of the data were collected in classrooms. As a result, an important population of students (students who miss class frequently) is missed; thus the level of student absenteeism seems to be even worse. Lastly, the data collection was not completed in a day; as a result, the authors cannot rule out the possibility of information contamination. Whereas, this study involved all students of medicine and health sciences and the therefore, there was little room for errors occurring by chance. The high response rate (89%) further strengthens the conclusiveness of this result.

The findings raise various issues that call for further research to determine better understand the reasons for absenteeism and its consequences. This trend should be taken seriously as it will have long-term consequences for the health-delivery and health services in Ethiopia.

## Conclusion

A significant number of study participants reported missing lectures in the previous semester. Missing lectures was associated with age of students, field of study, social drug use, lack of interest in the subject matter, and disliking the teaching style of the instructors. Cumulative grade point average, background of students, gender, parental education level, monthly allowance and class-size were not predictors of absenteeism in the study. Not only student behavior, but also teacher attributes and teaching methodologies appear to play a role in student absenteeism from lectures. Student absenteeism at a wider level will have detrimental consequences on societal values, productivity and economy of our country. Paying attention to the reasons for absenteeism reported by students could help institutions respond accordingly.

## Recommendation

Our findings indicate that to reduce the incidence of absenteeism, medical students and faculty need to be made aware of the problem and its immediate and long term consequences for the students and society at large. It is important to remind students this during admission and throughout their enrollment in medical school. Discouraging social drug use should also improve motivation to learn and increase attendance. In addition, higher institutions need to improve quality of lecture materials and teaching techniques to reduce absenteeism. Regular training for instructors to help them improve their teaching methodologies could be a useful intervention. In-depth investigation of reasons for missing classes using qualitative methods is warranted.

## Competing interests

The authors declare that they do not have any competing interests.

## Authors’ contributions

AAD conceived the idea, drafted the proposal and involved in all implementation stages of the project and write up; AB reviewed the proposal, and involved in all implementation stages of the project and write up. YB assisted the analysis and reviewed the proposal and the final manuscript. All authors approved final version of the manuscript.

## Pre-publication history

The pre-publication history for this paper can be accessed here:

http://www.biomedcentral.com/1472-6920/14/81/prepub
